# The Use of Community Engagement in Implementation: Successes and Challenges From the Implementation and Adaptation of Bundled HIV Interventions for Black Women

**DOI:** 10.1111/hex.70465

**Published:** 2025-10-24

**Authors:** Linda Sprague Martinez, Melanie Rocco, Judith C. Scott, Alex Bergson, Madison Kitchen, Andrea Dakin, Masill Miranda, Alicia Downes, Serena Rajabiun, Angela Wangari Walter

**Affiliations:** ^1^ Health Disparities Institute, UConn Health Hartford Connecticut USA; ^2^ Boston University School of Social Work Boston Massachusetts USA; ^3^ AIDS Foundation of Chicago Chicago Illinois USA; ^4^ AIDS United Washington District of Columbia USA; ^5^ Department of Public Health, Zuckerberg College of Health Sciences University of Massachusetts Lowell Lowell Massachusetts USA

**Keywords:** community engagement, HIV care, HIV intervention, implementation

## Abstract

**Introduction:**

Black women are disproportionately impacted by HIV in the United States, yet Black women are rarely called upon to engage in intervention development, implementation and evaluation. As a result, interventions continuously fail to reflect their priorities and needs. Incorporating the meaningful involvement of Black women with HIV and community organisations that serve them into implementation processes is necessary if interventions are going to be effective and sustained. The purpose of this paper is to examine the implementation strategies that employed community engagement to support the adaptation and uptake of bundled interventions to improve care and treatment outcomes for Black women with HIV as part of the Black Women First initiative. Successes and challenges in the use of community engagement in implementation are also explored.

**Methods:**

For this paper, we draw on qualitative implementation data collected over the course of the Black Women First initiative from 12 demonstration sites. Data‐related community engagement, patient, client, consumer engagement, implementation and challenges to engagement were extracted and analysed using directed content analysis.

**Results:**

We identified five types of community engagement used by sites in implementation: client advisory boards, client focus groups and surveys, peer leadership, community taskforces, and network weaving. They engaged site clients who were Black women with HIV, community members and partner organisations to inform pre‐implementation design, adaptation of intervention design and delivery, and intervention and programme sustainability planning. The frequency of use of these different types of community engagement varied across sites and the project period. Maintaining engagement and quality of involvement in implementation across the different types of community engagement were key challenges for sites, especially in advisory boards and focus groups.

**Conclusion:**

Researchers and HIV care practitioners should continue to utilise community engagement to enhance intervention implementation, as well as identify emergent ways to engage community members and organisations to enhance implementation and health outcomes for people with HIV.

**Public Contribution:**

Black Women First initiative demonstration site staff who were integral to the implementation of the bundled interventions for Black women with HIV were involved in the analysis and interpretation of the data, and preparation of this manuscript.

## 1 Introduction

Black women are disproportionately impacted by HIV in the United States and were identified as a priority population for the National HIV/AIDS Strategy (2022–2025) for ending the HIV epidemic [1, 2, 3]. The disproportionate burden of HIV experienced by Black women is driven by structural racism and the result of living in a society stratified by race, gender and social class [4]. This leaves Black women exposed to harmful conditions with limited access to health‐supporting resources and care [4, 5]. Moreover, Black women have historically been exploited and oppressed by the healthcare system, which further fuels mistrust [5]. Hence, there is a critical need to elevate Black women living with HIV's voices and priorities if we are to eliminate HIV inequities.

Community engagement (CE), meaningful participation and collaboration can help rebuild and restore trust [6]. Despite the documented benefits of involvement, Black women are rarely called upon to engage in intervention development, implementation and evaluation; thus, interventions continuously fail to reflect their priorities and needs [7, 8]. Comprehensive multilevel interventions that meaningfully involve diverse Black women and address the factors that produce and sustain HIV inequities they experience are needed if interventions are to be effective and sustained [1, 7, 8].

In this paper, we draw on qualitative implementation data from the Black Women First (BWF) initiative [9], a multi‐site national study of the adaptation and replications of bundled evidence‐informed interventions across 12 sites in the United States. This study examines the implementation strategies that employed CE to support the adaptation and uptake of the selected bundled interventions to improve care and treatment outcomes for Black women with HIV in their local area. CE is ‘the process of working collaboratively with and through groups of people affiliated by geographic proximity, special interest, or similar situations to address issues affecting the well‐being of those people’ [10]. A key CE tenet is that people in communities are the experts of their own lived experience and have a nuanced understanding of the factors impacting their health and well‐being [10, 11, 12]. CE is, thus, built on the premise that engaging community members impacted by inequitable outcomes and including them in decision‐making will lead to effective and sustainable solutions in public health [10, 13].

CE enhances community power, encourages the development of interventions tailored to the local context, improves research quality, develops sustained collaborations and leads to capacity building to address community‐identified priorities [14]. Moreover, as an evidence‐based practice [15], CE has been described as a vehicle to advance research translation [16] and catalyse the collaboration needed to expand access to novel treatments and reduce health care inequities [13, 15, 17]. CE can also help counter the deleterious effects of traditional top‐down approaches that discourage participation among minoritised groups [18]. This is because CE challenges dominant ideologies, which overlook the lived and living experience of minoritised populations. Thus, CE is an important tool for dismantling oppressive systems that stymie the meaningful involvement of Black women with HIV. However, CE approaches are iterative [19] and fall along a dynamic continuum [20]. Along the continuum, the level of involvement can range from outreach, such as connections with community organisations or members to share information, to shared leadership, or involving community organisations or members in all phases of implementation and dissemination as shared decision‐makers [20, 21]. Operationalising CE in the context of implementation may facilitate the meaningful involvement of Black women with HIV and advance the adaptation and implementation of bundled interventions. We explore how CE is employed in implementation strategies to address barriers and facilitators associated with the uptake and sustainment of bundled interventions that improve care for Black women with HIV and address health inequities. Findings illustrate how CE is used by demonstration sites to adapt and implement bundled interventions to improve the care for Black women with HIV. Successes and challenges in the use of CE in the overall implementation of bundled interventions are also discussed.

## 2 Methods

The overall evaluation protocol has been published elsewhere [9] and was reviewed by the University of Massachusetts Lowell and the Boston University Charles River Campus Institutional Review Boards, protocol numbers 20‐147 and 5832X. Mixed methods were employed to evaluate the implementation of evidence‐informed bundled interventions within and across the demonstration sites. A detailed description of the bundled interventions has been published elsewhere [6]. For this initiative, demonstration sites (*N* = 12) included community‐based organisations, public health departments and health centres across the United States [7, 9, 22]. As part of the evaluation framework, meaningful involvement of women was a noted implementation strategy and approach for the goal of the initiative [9, 23]. For this paper, we draw on qualitative implementation data collected over the course of the project (2020–2023) by the Evaluation and Technical Assistance Provider (ETAP). Table 1 provides a detailed description of each data source.

**Table 1:** Description of qualitative data sources for the BWF Initiative.

Data collection method	Description
Site Grant Applications (*n* = 12)	Funded site proposals submitted to HRSA during the Request For Applications (RFA) period.
Pre‐Implementation Team Interviews (*n* = 12)	Virtual interviews conducted by ETAP site coaches with demonstration site implementation staff and leadership at the beginning of the project. Interviews took place over two sessions and lasted approximately 2 h on average. All interviews were recorded and transcribed.
Monthly Call Forms (*n* = 199)	Ahead of monthly monitoring calls held between ETAP site coaches and the site, implementation staff completed a form summarising implementation updates, challenges and topics to discuss during their calls.
Annual Site Visit Reports (*n* = 24)	Annual site visits were conducted by ETAP staff. Site visits were facilitated by ETAP site coaches using a standard agenda. Site visit attendees include: implementation team members, site leadership and ETAP staff. Site visit notes were synthesised by ETAP staff in the form of a report, which was reviewed by the site. Site visit reports included: status updates on implementation processes, site‐level successes and challenges, training and technical assistance needs, and an HRSA fiscal and administrative review.
Post‐Implementation Individual Interviews (*N* = 76)	Individual interviews were conducted with implementation staff (*n* = 59) and community partners (*n* = 17). Community partners were identified for interviews by demonstration site staff based on duration and level of involvement in implementation. Interviews explored site perspectives on successes, challenges and lessons learned from the initiative. The average duration of interviews was 60 min. All interviews were recorded and transcribed.

### 2.1 Analytic Methods

Pre‐ and post‐implementation interview transcripts, monthly call forms, and site visit reports were uploaded to NVivo version 12.0 (Lumivero, Denver, Colorado) qualitative software and analysed using directed content analysis [24]. The code book was developed drawing on the Expert Recommendations for Implementing Change (ERIC) project [25], which created a common language for implementation strategies and encourages their consistent use [23]. There are a number of ERIC implementation strategies which employ CE, such as patient, client, consumer and community advisory boards and patient, client, consumer and staff feedback tools. Patients/clients, community members and community organisations can play different roles depending on the type of CE being employed in implementation and therefore varying levels of involvement along the CE continuum.

To better understand the types of CE used in implementation strategies, we analysed data from the codes for CE, patient, client, consumer engagement, implementation and challenges to engagement, which were included in the codebook and were the primary codes for this analysis. A copy of the codebook, which includes the codes and their definitions, is available on request. Three members of the research team coded transcripts, forms and reports (M.R., A.B. and M.K.). Coders read through transcripts and documents in NVivo and assigned text segments to codes that reflected definitions described in the codebook. Coders met throughout the process to reconcile codes and memos. During each meeting, intercoder reliability was determined by merging coded datasets and assessing agreement. In cases where agreement was below 80%, coders discussed and reconciled codes through discussion. In cases where questions remained or new codes emerged, the discussion was brought to meetings with the qualitative lead (L.S.M.) and project director (M.R.). These meetings were held weekly and focused on overall themes in the data, in addition to discrepancies and emergent codes. When coding was complete, reports on each code were summarised and reviewed by the research team to identify larger narratives within the data. Text segments illustrating primary themes were then selected. Descriptions of the types of CE described in site grant applications were reviewed to contextualise our findings.

## 3 Findings

Qualitative data revealed five distinct types of CE embedded in implementation strategies used by sites: client advisory boards (CABs); client focus groups and surveys; community taskforces; peer leadership; and network weaving. Sites were encouraged to use CABs, and the ETAP provided technical assistance to support the development and training of members as an implementation strategy for the uptake and replication of the bundled interventions.

As seen in Table 2, CABs, peer leadership and network weaving were the most commonly used types of CE across sites, and all sites used at least two unique types of CE in their implementation strategies. For some demonstration sites, the use of CE in programme implementation existed before the introduction of the BWF initiative. Illustrative quotes are provided to explore the role of CE in implementation in the next section.

**Table 2:** Type of community engagement used by BWF demonstration sites to enhance implementation.

Type of community engagement used in implementation	Definition of type	# of sites that utilised type
Client Advisory Boards (CABs)	Client advisory boards brought current and/or former site clients who are Black women with HIV into key decision‐making roles to inform implementation [23].	11
Client Focus Groups and Surveys	Focus groups and surveys administered to former and current site clients who are Black women with HIV to inform bundled intervention design and implementation.	6
Peer Leadership	Hiring Black women with HIV in client‐facing staff and leadership roles as part of the bundled intervention programme at the given demonstration site. This does not include peer‐based interventions included as part of BWF sites' bundled interventions.	11
Community Taskforces	Groups that brought together local partners and champions to improve implementation and streamline access to care for Black women with HIV.	2
Network Weaving	Network weaving is the process of both building on existing relationships and developing new ones within and outside of the organisation to promote information sharing that supports implementation [23]. Network weaving was a strategy used both internally and externally by the organisations.	12

### 3.1 CE Integrated in Implementation

As seen in Table 3, CE was utilised by sites throughout the implementation processes. Challenges and lessons learned about the use of CE in implementation strategies are also explored.

**Table 3:** Type of community engagement used in each implementation process.

Type of community engagement	Implementation process			
	Pre‐Implementation	Implementation	Fidelity monitoring and adaptation	Sustainability planning
Client Advisory Boards	X	X	X	X
Client Focus Groups and Surveys	X	X	X	
Peer Leadership		X	X	
Community Taskforces	X	X		X
Network Weaving	X	X	X	X

#### 3.1.1 Pre‐Implementation

Sites used three forms of CE during the pre‐implementation process: CABs, client focus groups and surveys, and outreach and network weaving. CABs were employed by most demonstration sites and met regularly over the course of the funding period. In some cases, sites leveraged existing boards at their organisations and encouraged more participation of Black women with HIV. During pre‐implementation, boards informed the intervention design and advised on hiring and implementation planning.

*Early on in the program, CAB members participated in a pilot of the [name] program so that they could experience the program being implemented with the changes they had previously proposed incorporated into the design*.

*The Patient Leadership Advisory Group (PLAG) is comprised of staff and clients who have a stake in the implementation of all new services at the clinic. PLAG members were key to identifying staff to be hired for this project and in the design of [program name]…*

Client focus groups and surveys were used during pre‐implementation to ensure client voices were represented in intervention design and implementation. Groups and surveys were employed during the pre‐implementation phase as well as to inform the monitoring and adaption phase.

*The target population was involved in designing the proposed program, sharing information about community needs and suggestions for program flow, through 5 different focus* groups *and 5 key informant interviews. Further, the Evaluator will conduct a participatory evaluation process, including Black women with HIV throughout the process including in design of data collection tools, sharing information on community needs throughout the program, and identifying ways and questions to pose during qualitative evaluation activities (focus groups, surveys and interviews)*.

The third approach used during the pre‐implementation phase was network weaving. We found that organisations used network weaving to inform their interventions and plan for intervention delivery. In some instances, this involved identifying and coordinating with partner organisations to implement elements of the bundle.

*[Site name]and their* partners *will deliver the intervention at their sites in person and virtually as necessary. There is space at each site to conduct 1‐1 and group interventions. The 3 bundled interventions were designed to be adapted to the partner agency workflow*.

#### 3.1.2 Implementation

During the implementation process, sites used all five CE approaches. Sites describe the role of advisory boards in implementation.

*Beyond that, they will be engaged in the implementation of the bundled evidence‐informed intervention by providing feedback on the project development, implementation and evaluation. The advice of the Board will drive all decision‐making activities* pertaining *to the implementation of the project*.

Focus groups and feedback tools used during pre‐implementation were also included early on in implementation to inform initial implementation processes.

*Focus groups with the priority population have provided feedback on recruitment and best practices to engage women who are newly diagnosed. Their feedback has focused on outreach*, *recruitment, and initial implementation*.

Meanwhile, community taskforces made up of community leaders, local organisations, partners with population‐specific expertise, and peers were engaged in implementation. Their involvement often involved training and real‐time advising on implementation practices.

*Taskforce* Structural *Overview: Expand capacity of organizations to work in and with reentry…. Provide trainings on stigma reduction, TIC (trauma‐informed care), SUD (substance use disorder), and MH (mental health) with providers…. Ensure the reentry population is a part of the taskforce, as well as a part of workgroups and advising on programs*.

The inclusion of peers was a common form of CE used across sites during the implementation phase. Peers delivered interventions such as educational components and resource navigation and informed intervention design. Specifically, they provided intervention related trainings, outreach and engagement. Sites described the critical role of peers in enhancing the delivery of the bundle and expanding services to reach diverse groups of Black women.

*Our strong peer support program, which has allowed them to increase their reach. Through word of* mouth *and peers, [site name] has expanded their reach among Black women and is now engaging diverse Black immigrant and Transwomen*.

Network weaving was also described by sites as a form of CE used during implementation. Sites networked within their organisation across departmental units as well as with a wide variety of external organisations to promote the bundle as well as integrate it into existing programming.

*An additional* strength *of the [program name] is the way in which it has been promoted and marketed across [site name]. The team has been intentional in the ways in which it has engaged programs across the agency, such as attending interdepartmental meetings to introduce services, as well as conducting smaller meetings with case management teams to provide a more detailed description of services. Additionally, they invited other programs to outreach events to describe their services and provide wellness workshops. This has contributed to awareness of the programs as well as program reach*.

*To support the implementation of these bundled interventions, [site name] is collaborating with partner agencies … to conduct staff trainings and agency capacity building to enhance* service *delivery to clients in needs of specific services*.

#### 3.1.3 Monitoring and Adaptation

CE was also prominent during the monitoring and adaptation processes of implementation. Sites described using four of the five forms of CE in these processes: advisory boards, focus groups, surveys and network weaving.

CAB feedback was used by sites to adapt interventions throughout the implementation period. They reflected on implementation processes and provided feedback on programme design and materials that led to adaptations.

*…they had a group of* women *who were chosen to be part of it [board name] and we had one of the case managers … there to monitor and help with that. They were meeting on a … monthly basis to talk about things related to the program, changes that need to take place and so on and so forth. And then when we have our team meeting, she (the case manager) will report back…*

*So, when there's something that they're not pleased with, that's where the CAB comes in when it comes to how the program functions with things like incentives and the CAB can say, no, this does not serve us. Can we do something different? And if it's within my realm of changing, I can do that. There's an event coming up—what would you like to see? It is about allowing people to utilize and amplify their own voices without you telling it for them…*

Focus groups, surveys and peer leadership were similarly used to inform adaptations made to the bundled interventions.

*A survey to elicit feedback from the first cohort of [program name] enrollees, was administered to determine if the program/modules delivery format required any modification, which was then* modified *accordingly for the next cohort of women/enrollees*.

*So, it's supporting leadership training, supporting these events to have peer leadership in and guide and these specific conferences that come out of that and come up that will address more the need in DV (domestic violence) services for clients, in this case, Black women, women of color, or other needs that they might have. But it comes from giving that support to leadership to have the time and develop the skills so they can think back and help guide that process*.

Finally, network weaving with partner organisations allowed sites to further tailor and adapt interventions as well as to fine‐tune implementation processes.

*Now that the recruitment process has begun, we will continue to work with our partnering HIV providers to fine‐tune recruitment strategies to best meet the needs of this interventions and align with their clinic workflow*.

*To support the implementation of these bundled interventions, [Site name] is collaborating with partner agencies … to conduct staff trainings and agency capacity building to enhance service delivery to clients in needs of specific services*.

#### 3.1.4 Sustainability Planning

CABs, community taskforces and network weaving were all used to inform sustainability planning. In the case of advisory boards and taskforces, sites described the value of engaging with the board to inform sustainability planning and identifying what elements of the BWF site programme should be sustained, as well as sustaining the board itself.

*[Site name] recognizes the value of having a CAB and hopes to sustain efforts to support recommendations for other [site name] RWHAP. Additionally, the team expects to expand CAB membership post [program name] to represent all client demographics served by [site name]*.

…*It (training) was very helpful to gather information to move forward with the CAB. We created a plan to expand the CAB to include opportunities to garner more feedback about sustainability*.

Networking weaving was another form of CE used for sustainability planning.

*…we're still working to partner with other organizations and continue to do that [plan for sustainability]. [partner organization], working with those apartment complexes to make sure that at least they're getting their apartments filled, and we are getting housing units for our ladies. We want to continue sustaining those things because it will help in the future with resources*.

*We (community partners and implementers) have monthly, every other month calls, and as we talk sustainability, we're always talking, and it's true, funding does come up, but we also talk about ways in* which *we can*.

## 4 Challenges and Lessons Learned

Demonstration sites participating in the BWF initiative faced various challenges when employing CE in implementation strategies, such as challenges with recruitment and engagement on boards and taskforces, and insufficient organisational capacity and funding to sustain CE, which may have impacted the implementation processes and sustainment. Sites reflected on their use of CABs and focus groups and what challenges they faced in recruiting and sustaining engagement.

*Things have slowed down with the CAB. It has been difficult to attract and retain interested parties.*

*In recruiting for the focus group, there was a heavy hand of, ‘Oh would you guys encourage your patients to do it?’ And it was like, ‘Yeah, sure’, but it's such a small population and you're selecting for people who are already engaged in care and already have these extra layers of support*.

Sites reported that CABs needed a clearly defined purpose and structure to be most successful. Without this definition, they reported having a difficult time keeping Black women with HIV engaged and ensuring their perspectives informed implementation.

*I mean, one of the things that's hard is if you're pulling a CAB together, we probably should have been clear about who could be in the CAB and that kind of thing. Because you only have a hundred clients essentially, so they're actually not the best people to be on the cab*, *some of them, because their lives are so, chaotic*.

*There're many things that I know that could have been done differently. Some of the things that I* thought *probably would have been implemented is having the women have goals that needed to be set, desires that they would want for their organization*.

Additionally, some CABs were not BWF initiative‐specific and rather site‐wide, resulting in advisory boards that may not have included Black women with HIV or their perspectives on implementation of the BWF bundled interventions.

*I think there should be more women on the advisory board, especially women of color. Because we have a perspective, we have a very colorful perspective. But other than them including us and* listening *to us, because once you include us and you listen to us, they'll hear something then the conversation would change*.

There were also challenges related to recruitment and sustained engagement for sites that utilised community taskforces. Sites reported that, for community taskforces to be influential in implementation, there needed to be a clear purpose and ongoing communication.

*I think the biggest* challenge *was helping them understand that this is a task force for you, and what you put in it is what you're going to get out of it. So, I really had to work on … allowing them to understand that because we were meeting every other month and my biggest thing for them was, this is your network. So, just because we're not sitting in a meeting with each other doesn't mean that you watch shouldn't be communicating…. It doesn't work if you don't work it. So outside of meetings outside of meetings you all should still be communicating*.

At different times throughout the project period, site staff mentioned wanting to have more peer roles on the programme team because they recognised the value of peers in implementation, but didn't have the funding or capacity to do so.

*I was saying a peer navigator I think that would be beneficial. If possible, to fully fund, I guess the care coordinator position or peer navigator because I've noticed that the ladies need to be able to engage with someone frequently. And, also, to have more offices to have peers, especially if they're wondering why clients are not coming, but to have somebody that's either a peer or community health worker that's living with HIV and to see how that makes an impact on clients that there's* somebody *like them that they can talk to*.

In their outreach and network‐weaving activities, sites faced similar challenges with recruitment and sustaining relationships with organisational partners.

*Despite developing important marketing materials and hosting events, partnerships with other agencies in the [site city] area is a challenge. Very few participants are enrolled in the program which is not in alignment with the high incidence and prevalence of HIV among Black women in this region.*

A demonstration site staff member reflected on how the lack of partnerships potentially resulted in further work for the implementing team.

*I think that that was a huge, missed opportunity, because of the fact that there are already organization(s) that been doing this work for years and are clearly very effective at it. I think we wouldn't have had to create some of the wheels that we tried to create, even though them wheels are kind of rough. We could have learned a lot from them in terms of the things we could have provided to our…. The little things like that, that could have shown a little bit more investment into the people that we were serving were some missed opportunities*.

## 5 Discussion

In this paper, we set out to describe how CE is used in the implementation of bundled interventions to improve care for Black women with HIV. In doing so, we identified five types of CE used by sites in their implementation processes: CABs, client focus groups and surveys, peer leadership, community taskforces and network weaving. Through these CE approaches, sites engaged clients who were Black women with HIV, community members and partner organisations to inform pre‐implementation design, adaptation of intervention design and delivery, and intervention and programme sustainability planning. In each implementation process, the frequency of the type of CE used varied. For example, sites facilitated focus groups or disseminated surveys, which are often used to gain community input [26, 27], to inform the monitoring and adaptation of implementation strategies, whereas advisory boards and peer leaders were a type of CE that was ongoing in monitoring and adaptation. Maintaining engagement and quality of involvement in implementation across different forms of CE was a key challenge for sites, especially in the client‐facing CE, such as advisory boards. The literature indicates that sustaining engagement over time is a challenge, given the extensive time commitment and the many competing interests patients are confronted with [28].

The use of CE by BWF demonstration sites is consistent with the literature regarding the role of CE in the implementation of interventions, especially HIV care interventions. CE is a critical component in bridging the evidence‐to‐practice gap integral to implementation science [29]. Community partners and individuals with lived experience possess in‐depth knowledge of their local communities' strengths and needs, which can be applied to the integration and adaptation of interventions to improve acceptability, feasibility and relevance [29]. In the context of HIV care, CE, such as advisory boards, has been associated with positive HIV‐related outcomes [30]. CE employed by BWF demonstration sites provided opportunities for Black women with HIV to inform programme design, hiring, intervention monitoring and evaluation.

While this study contributes valuable insights into how CE can inform the implementation of bundled intervention programmes, there are several limitations. Firstly, most of the data used in our analysis comes from implementation site staff. Community partners were only directly engaged in post‐implementation individual interviews. As a result, valuable insights and lessons learned about CE from the perspective of community partners are limited. Secondly, outcomes of the use of CE in implementation processes were not measured by the BWF ETAP. As a result, we cannot determine the extent to which engagement strategies were effective or meaningful in implementation or health outcomes for Black women with HIV. Future research should explore how implementation science can better incorporate and measure engagement along the spectrum of involvement, moving beyond the assumption that engagement strategies inherently work. Thirdly, this study relied on predefined, evidence‐based engagement strategies from the literature, which may have overlooked other meaningful ways that communities, peers and clients were engaged in implementation. Therefore, we may have missed opportunities to document organic or emergent forms of engagement that also enhanced implementation processes. Future research should consider how to conceptualise engagement in its multiplicity, recognising the diverse ways in which communities participate in and influence implementation efforts. By addressing these limitations, future research can deepen our understanding of how engagement operates in implementation science, moving towards more nuanced and community‐driven approaches.

## 6 Conclusion

Authentic and sustained CE in implementation is needed to understand complex drivers of inequities and create solutions that advance health equity in an inclusive way [31]. Researchers and HIV care practitioners should continue to utilise CE to enhance intervention implementation, as well as identify emergent ways to engage community members and organisations to enhance implementation and health outcomes for people with HIV. Funders should also consider integrating CE approaches and strategies into the design of requests for applications.

## Author Contributions

**Linda Sprague Martinez:** conceptualization, methodology, original writing, review and editing, supervision. **Melanie Rocco:** conceptualization, data analysis, original writing, review and editing. **Judith C. Scott:** review and editing. **Alex Bergson:** data analysis, review and editing. **Madison Kitchen:** data analysis, review and editing. **Andrea Dakin:** review and editing. Masill Miranda: review and editing. **Alicia Downes:** review and editing. **Serena Rajabiun:** funding acquisition, review and editing. **Angela Wangari Walter:** conceptualization, review and editing.

## Disclosure

The opinions and views expressed herein are those of the author(s) and do not represent the official policies of, nor an endorsement by, Health Resources and Services Administration, HHS or the U.S. Government.

## Ethics Statement

Ethics approvals were obtained from the University of Massachusetts Lowell and the Boston University Charles River Campus institutional review boards, protocol numbers 20‐147 and 5832X.

## Conflicts of Interest

The authors declare no conflicts of interest.

## Data Availability

The data that support the findings of this study are available from the principal investigator and project contact Serena Rajabiun upon reasonable request.
